# Editorial: The role of gut microbiome metabolites in cardiometabolic disorders

**DOI:** 10.3389/fnut.2026.1759753

**Published:** 2026-01-19

**Authors:** Mateusz Szudzik, Antoni Sureda, Monica Barone

**Affiliations:** 1 Laboratory of Centre for Preclinical Research, Department of Experimental Physiology and Pathophysiology, Medical University of Warsaw, Warsaw, Poland; 2 Research Group on Community Nutrition and Oxidative Stress, University of the Balearic Islands-IUNICS, Health Research Institute of Balearic Islands (IdISBa), Palma, Spain; 3 CIBER Fisiopatología de la Obesidad y Nutrición (CIBEROBN), Instituto de Salud Carlos III (ISCIII), Madrid, Spain; 4 Human Microbiomics Unit, Department of Medical and Surgical Sciences, University of Bologna, Bologna, Italy

**Keywords:** bacterial metabolites and co-metabolites, cardiometabolic disease, cardiovascular, SCFAs, microbiome

## Introduction

Growing evidence shows that the gut microbiome is not a passive passenger but an active metabolic “organ” producing bioactive compounds that shape systemic metabolic homeostasis and cardiovascular function. Microbiome-derived metabolites, including short-chain fatty acids (SCFAs), bile acids, branched-chain amino acids, and tryptophan metabolites, exert a profound influence on inflammation, energy regulation, endothelial function, and neurocardiac communication.

Cardiometabolic diseases remain a leading global health challenge, and traditional risk factors such as diet, lifestyle, and genetics do not fully explain the variability in disease onset and progression—even under similar environmental conditions and with comparable genotypes (1). Microbiome-derived metabolites may represent a missing mechanistic link, operating along multiple systems within the human body, a concept increasingly described in terms of specific *gut–target organ* axes, including the gut-heart, gut-metabolic, and gut-brain axes. These pathways modulate host physiology at molecular, cellular, and organ levels, affecting inflammation, glucose and lipid metabolism, intestinal barrier integrity, and receptor-mediated signaling (2, 3).

## Scope of this Research Topic

This Research Topic assembles six complementary reviews that examine the microbiome–cardiometabolic interface from mechanistic, translational, and clinical angles. Together, they illuminate how microbial metabolites influence acute cardiac injury, chronic cardiometabolic diseases, glucose regulation, drug metabolism, and psychocardiological interactions.

## Collection of reviews

### Gut-hearth axis and ischemia/reperfusion injury

Chen et al. explore the involvement of the microbiome in myocardial ischemia/reperfusion (I/R) injury. Their review highlights intestinal barrier dysfunction, inflammatory activation, oxidative stress, and mitochondrial impairment as key components linking gut dysbiosis to cardiac damage. Importantly, they summarize microbiota-targeted strategies—including high-fiber diets, SCFA supplementation, probiotics, and traditional Chinese medicine that may mitigate reperfusion injury.

This article underscores the emerging concept that the microbiome can serve as a therapeutic target not only in chronic cardiovascular conditions but also in acute cardiac events.

### Microbiota–metabolite interactions in diabetic cardiomyopathy

Ji et al. review how gut dysbiosis contributes to diabetic cardiomyopathy (DCM). They detail the roles of SCFAs, bile acids, and branched-chain amino acids influence insulin resistance, oxidative stress, cardiac inflammation, and remodeling. Their discussion of therapeutic strategies, including microbiota modulation, fecal microbiota transplantation (FMT), probiotics/prebiotics, and other approaches, provides a solid mechanistic basis for future translational studies aimed at shifting metabolite profiles in a way that counteracts cardiac remodeling, fibrosis, and deterioration of systolic function, preventing or slowing DCM progression.

This article provides a solid theoretical and mechanistic basis, pointing to directions that can be developed clinically in the pathology discussed.

Together, these first two reviews emphasize how gut-heart communication contributes to both acute and chronic cardiac pathology.

### Butyrate as a multifunctional regulator in cardiovascular diseases

Xu et al. conducted a review of both basic and clinical studies focused on the SCFA butyrate and its diverse signaling pathways, including G-protein coupled receptor (GPCR) activation, histone deacetylase (HDAC) inhibition, and peroxisome proliferator-activated receptors (PPARs) modulation. They describe its associations with hypertension, atherosclerosis, coronary artery disease, atrial fibrillation, heart failure, obesity, and diabetes. The review also considers the therapeutic potential of direct butyrate administration and dietary strategies to enhance endogenous production, while discussing key translational challenges.

### Butyrate and glycemic control

Hamari et al. present a semi-systematic review of butyrate's role in glucose homeostasis from both animal and human studies. They discussed evidence that microbiome-derived butyrate may improve insulin sensitivity, reduce inflammation, and modulate body weight. Critically, they address methodological inconsistencies across studies—differences in butyrate delivery, absorption variability, and individual differences in endogenous production—and evaluate dietary interventions (various fibers), such as sodium butyrate, butyrins, acylated starch, prebiotics, and postbiotics. While findings are promising, they conclude that controlled clinical trials are needed to determine optimal strategies for increasing butyrate levels in humans.

These two (Xu et al.; Hamari et al.) butyrate-focused reviews together underscore the centrality of SCFAs in cardiometabolic regulation and highlight the translational opportunities and challenges of targeting microbial metabolites. Butyrate may not only be a “beneficial metabolite” produced in healthy individuals, but also an active mediator of vascular health with potential therapeutic use during the disease.

### Bidirectional interactions between cardiovascular drugs and the gut microbiota

Wang et al. analyze the bidirectional interactions between the gut microbiota and commonly used medications in the treatment of CVD. They describe mechanisms through which the gut microbiota metabolizes cardiovascular drugs (e.g., via bacterial enzymes), thereby influencing their bioavailability, efficacy, and side effects. Conversely, cardiovascular medications reshape microbiota composition. This bidirectional view reveals potential for microbiome-aware, personalized pharmacotherapy.

This is a highly practical article: it highlights that personalization of pharmacotherapy in cardiovascular diseases may consider the state of the microbiome, which is crucial for both drug efficacy and safety.

### The gut-hearth-brain axis and psychocardiology

Lai et al. examine the interplay between mental health disorders (e.g., depression and anxiety that co-occur with cardiovascular diseases) and cardiovascular disease through the lens of the gut-hearth-brain axis. They highlight the role of gut microbiota and its metabolites (such as SCFAs, indole derivatives, and tryptophan metabolites) in neuromodulation and cardiovascular regulation. Their work suggests that microbiota modulation may represent an innovative therapeutic approach to conditions at the intersection of psychology and cardiology. Because the microbiome is involved in producing key neuromodulatory compounds, strategies targeting it may benefit both mental and cardiovascular health.

## Summary and future perspectives

Collectively, these six reviews demonstrate that gut microbiome–derived metabolites are active mediators playing a multifaceted role in the pathogenesis and potential treatment of cardiometabolic diseases. Acting through receptor signaling, epigenetic modulation, immune regulation, and metabolic pathways, these metabolites influence inflammation, tissue remodeling, mitochondrial function, and cardiac performance.

### 1 . Need for Translationally Oriented Basic Research

To accurately capture the full complexity of host-microbiome-metabolite interactions within cardiometabolic disorders, future interdisciplinary experimental designs should integrate physiologically relevant animal models, advanced cell systems, and controlled perturbation studies aimed at examining the full spectrum of potential interactions. At the same time, basic research should ensure translational relevance and mirror the complexity of human cardiometabolic physiology, enabling the closest possible approximation of processes occurring in patients.

### 2 . Clinical Trials and Standardization

Robust, randomized, long-term clinical trials are required to establish the efficacy and safety of microbiome-targeted therapies. Standardized methodologies for measuring key metabolites and microbiome endpoints will be essential to harmonize data across research centers and build coherent clinical conclusions.

### 3 . Multi-omics and Computational Integration

A crucial future direction involves integrating microbial metagenomics, host transcriptomics, metabolomics, and clinical phenotyping. Machine learning-based analyses may help identify novel metabolite signatures and biomarkers predictive of disease risk or therapeutic response, enabling more precise, personalized cardiometabolic interventions.

### 4 . Emerging Therapeutic Approaches

The field is rapidly evolving toward engineered probiotics, targeted postbiotic formulations, synbiotics, and dietary strategies designed to modulate metabolite production. Equally important will be addressing interindividual variability in microbiome composition and metabolite generation, which remains a major barrier to clinical translation.

## Conclusion

Altogether, this Research Topic illustrates the transformative potential of microbiome science in reshaping our understanding—and future treatment—of cardiometabolic diseases. We hope these contributions stimulate new interdisciplinary collaborations bridging microbiology, cardiology, metabolism, neuroscience, and personalized medicine.
